# *QuickStats*: Percentage* of Adults Aged ≥18 Years Who Met the Federal Guidelines for Muscle-Strengthening Physical Activity,† by Age Group and Sex — National Health Interview Survey, United States, 2020§

**DOI:** 10.15585/mmwr.mm7118a6

**Published:** 2022-05-06

**Figure:**
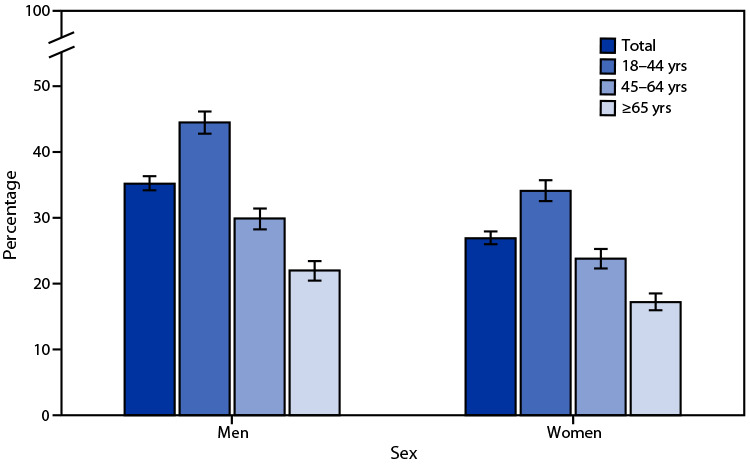
In 2020, 35.2% of men and 26.9% of women aged ≥18 years met the federal guideline for muscle-strengthening physical activity. The percentage of men who met the muscle-strengthening guideline decreased with age from 44.5% for those aged 18–44 years, to 29.9% for those aged 45–64 years, and to 22.0% for those aged ≥65 years. The percentage also decreased with age among women from 34.1% for those aged 18–44 years, to 23.8% for those aged 45–64 years, and to 17.2% for those aged ≥65 years. Men were more likely to have met the muscle-strengthening guideline than women in all age groups.

**Source:** National Center for Health Statistics, National Health Interview Survey, 2020. https://www.cdc.gov/nchs/nhis.htm

